# An Augmented Perceptual-Cognitive Intervention Using a Pattern Recall Paradigm With Junior Soccer Players

**DOI:** 10.3389/fpsyg.2018.01260

**Published:** 2018-08-23

**Authors:** Jörg Schorer, Marlen Schapschröer, Lennart Fischer, Johannes Habben, Joseph Baker

**Affiliations:** ^1^Institute of Sport Science, University of Oldenburg, Oldenburg, Germany; ^2^Institute of Movement and Neurosciences, German Sport University Cologne, Cologne, Germany; ^3^Institute of Sport and Exercise Sciences, University of Münster, Münster, Germany; ^4^School of Kinesiology and Health Science, York University, Toronto, ON, Canada

**Keywords:** tactics, expertise, field study, video training, talent development

## Abstract

In sport, perceptual skill training software is intended to assist tactical training in the field. The aim of this field study was to test whether “laboratory-based” pattern recall training would augment tactical skill training performed on the field. Twenty-six soccer players between 14 and 16 years of age from a single team participated in this study and were divided into three groups. The first received field training on a specific tactical skill plus cognitive training sessions on the pattern recall task. The second performed only the field training while the third group served as a control group and had field training on other topics. The task on the pre-, post-, and retention-tests was to recall specific soccer patterns displayed on a computer screen. Results showed significant changes between pre- and post-test performance. There was no significant interaction between groups and tests but the effect size was large. From pre- to retention-test, there was a significant difference between tests and an interaction between groups and tests, but no main effect difference between groups. On the basis of significance testing only retention was affected by the additional training, however, descriptive results and effect sizes from pre- to post-test were as expected and suggested there were learning benefits. Together these results indicate that augmented perceptual-cognitive training might be beneficial, but some limitations in our study design (e.g., missing field test, missing placebo group, etc.) need to be improved in future work.

## Introduction

Starkes and Lindley (1994) considered whether the development of sport expertise could be hastened through the use of video simulations. While this sparked a variety of research that looked to improve performance in the lab, little research has considered whether field training could be augmented by using perceptual-cognitive interventions on a computer (for an exception see Christina et al., 1990). This was the focus of our investigation.

Considerable research attention has been given to understanding the role of video training in facilitating the training of perceptual-cognitive skills (Williams and Grant, 1999). Underpinning our understanding of expert perceptual-cognitive skill in sports is the robust research base emphasizing the malleability of these skills with appropriate training (Williams and Ford, 2008). Differences between expert athletes and lesser skilled performers have been identified in several areas of perceptual skill (for an overview compare Williams et al., 2011; Williams and Abernethy, 2012). Several studies have shown that skills like decision making (Put et al., 2013) and anticipation (Murgia et al., 2014) can be trained.

Perhaps most relevant for the current investigation, one of the most consistently noted skills has been the ability to recall patterns of domain specific information (cf. Williams and Abernethy, 2012). For example, in team-based interactive sports, experts have been shown to have superior recall of the offensive and defensive structure in their sport than lesser skilled performers (Farrow and Abernethy, 2015). Further, experts’ recall performance is only superior in domain specific structured tasks (e.g., Abernethy et al., 1994; Williams et al., 2004). This might be explained by the experts’ development of a detailed sport-specific memory of situations and strategies that they experienced during their practice and training (cf. Farrow, 2011). Based on a theoretical foundation from early studies of chess by de Groot (1965) as well as Chase and Simon (1973) and Simon and Chase (1973), expertise differences in pattern recall have been demonstrated in several sports like American football (Garland and Barry, 1991), basketball (Gorman et al., 2012, 2013), field hockey (Starkes, 1987), soccer (Williams et al., 1993; Williams and Davids, 1995; Ward and Williams, 2003), snooker (Abernethy et al., 1994), and volleyball (Borgeaud and Abernethy, 1987). Moreover, the transferability of pattern recall skill has been demonstrated in sports with similar patterns of defense or offense (Smeeton et al., 2004; Abernethy et al., 2005). Researchers have also investigated anticipatory perception in pattern recall tasks, suggesting experts apply an anticipatory encoding of information when solving pattern recall tasks (Gorman et al., 2012, 2013; van Maarseveen et al., 2015) and that this effect also occurs when a series of patterns is used that is shown right before and right after the target image (Gorman et al., 2017).

Despite the consistency of these findings, we know very little about how these skills are trained (Williams and Grant, 1999; Schorer et al., 2015). Previous studies of perceptual training in sport have focused on the influence of different forms of instruction (Smeeton et al., 2005; Abernethy et al., 2012) or feedback (Memmert et al., 2009; Schorer et al., 2010), as well as transfer from the laboratory to field settings (Scott et al., 1998; Williams et al., 2003; van Maarseveen et al., 2016) or from virtual realities to reality (Tirp et al., 2015).

While these studies provide insight into the conditions of perceptual training in the laboratory, they have not evaluated whether perceptual training is useful as an adjunct to normal field training (for exceptions see Christina et al., 1990; Singer et al., 1994; Abernethy et al., 1999; Williams et al., 2002; Gorman and Farrow, 2009). The aim of this study was to determine whether additional pattern recall training off the field is beneficial in combination with “normal” field training for the acquisition and retention of pattern recall skill. Our first hypothesis was that there should be a greater improvement for groups with augmented cognitive training in comparison to only field training and a control group. In our second hypothesis, we assumed the augmented training group would show better retention over time than the other groups. Retention tests are especially important in field studies to demonstrate the efficacy and long-term effects in learning studies (Williams and Grant, 1999; Schorer et al., 2015).

## Materials and Methods

### Participants

Twenty-six youth team male soccer players (mean age = 15.56 years, *s* = 0.93) participated voluntarily in this study. All played on a single team, in the second highest regional league for their age. All participants were randomly allocated to three different groups, which are described in more detail later. All reported normal or corrected-to-normal vision. Because the players were under age, their parents and the participants provided written informed consent before this study. The study was conducted in accordance with the revised ethical declaration of Helsinki.

### Stimuli

**FIGURE 1:**
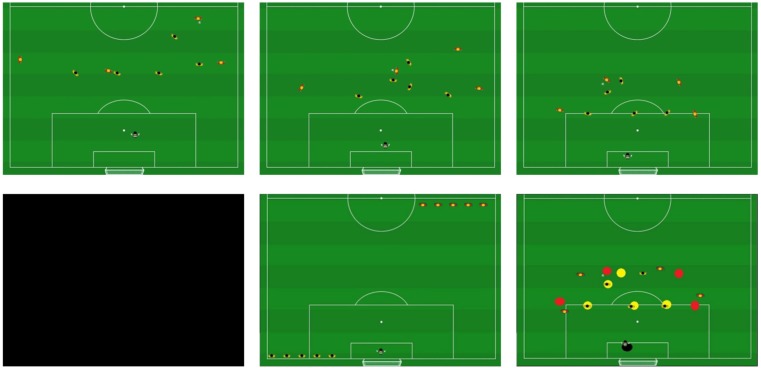
Schematic presentation of the pattern recall task. In row 1, a series of images shows the animations presented to the participants during the task. The last image is an example representing the “frozen” frame at the end of the animation. The black image (row 2, left image) was followed by a recall screen (row 2, middle image). During training players received feedback as demonstrated in row 2, right image.

For the task, animations presenting different soccer game situations were developed by two experienced coaches (cf. **Figure 1**). While a higher level of fidelity would have been reached by using real videos, they also raise methodological concerns. For example, in real videos the exact position of the presented player is not clear, because the position could be either his or her feet or the stomach or any other defined body part. On tactical boards such as the ones used here, the *x*- and *y*-axis position is clearly defined and therefore easy to measure. Moreover, this type of tactical display is very commonly used by coaches. The colored animations were compiled by the program Easy Animations 3.0 and included small yellow and red icons representing the soccer players. The experiment was programmed using Experiment Builder (SR Research) and the animations were presented from an aerial perspective showing one half of a soccer pitch. When the animations begin, the offenders leave the beginning player formation and start moving on the pitch, passing the ball to different players. The defenders shift their positions depending on, and adapting to, the attackers’ movements. The animations showed structured attacking situations with the defending team reacting by using typical structured back four defenses. Each scene contained five outfield players per team. The animated scenes had a length of 5 s with the last frame “frozen” for another 5 s followed by a black screen for 2 s. After each animation, a screen presenting the figures and the pitch appeared, which the participants used to position their recalled players. Participants used their forefinger to place the recalled players on the touchscreen (AcerZ5610).

### Procedure

#### Pre-test, Post-test, and Retention-Test

In each test, participants saw 10 evolving tactical animations as described above on a 23-inch touchscreen (Acer Z5610). The participant’s task was to replicate the player formation of the last still image of the presented video as precisely as possible. Using their forefinger as the cursor, participants were able to move the various player figures around the pitch. The time between pre- and post-test was 4 weeks with training twice a week. The retention-test was conducted 2 weeks after the post-test.

#### Field Training

This study was implemented during the normal training of a youth team. Field training consisted of normal elements of training including warm-up, technical drills, and playing football games. During the tactical training all players received instructions by a coach who was unaware of which player was in which group for the training study. The topic of the tactical training on the field was the same as in the stimuli presented in the animations (i.e., the back four defense). Field training was conducted twice per week for 4 weeks and lasted approximately 90 min.

#### Perceptual-Cognitive Training

Perceptual-cognitive training was also conducted twice a week for 4 weeks. Participants in this group performed training once before and once after the normal field training sessions per week. The task was the same as in the tests with the addition that, after recalling the positions of the players, participants received immediate feedback. Feedback was provided by yellow and red circles indicating the real position of the players in comparison to the recalled positions. Each training session lasted approximately 30 min and in each session, 14 out of 28 situations were randomly selected by the computer and presented in random order for each session and each participant. The scenes used in training were different from the test scenes.

#### Training Groups

In our study, three different groups participated:

(1) *Cognitive and field training group (n = 10).* The cognitive and field training groups participated in both forms of training described above.
(2) *Field training group (n = 10)*. This group participated only in the field training.
(3) *Control group (n = 6)*. The control group did not receive any training on this specific tactical situation, however, they participated in different forms of field training.

### Statistical Analyses and Dependent Measures

All data were analyzed using SPSS 22.0 and G-Power 3.10 (Faul et al., 2007). For data analysis, the dependent variable was minimized root mean square error (RMSE). Because our task did not assign players to specific positions, we calculated all possible configurations of distances between real and recalled player positions and used minimal distance as the dependent variable. We then ran two hypothesis-driven analyses. First, a mixed-model factorial analyses of variance was done with test (pre- to post-test) as the repeated measure and group as the factorial measure. Second, we conducted the same analysis, but with the repeated measure from pre- to retention-tests. Prior to these analyses, we ran a baseline check. Alpha was set at 0.5 and effect sizes were calculated as *f*-values (cf. Cohen, 1988). Values of *f* = 0.10 and above were interpreted as small, while values of 0.25 and above indicated a medium effect size and of 0.40 and larger indicated a large effect (Cohen, 1992).

## Results

In a first step, pre-test differences between groups were considered. This baseline check revealed no significant differences between groups, *F*_(2,25)_ = 1.53, *p* = 0.24.

**FIGURE 2:**
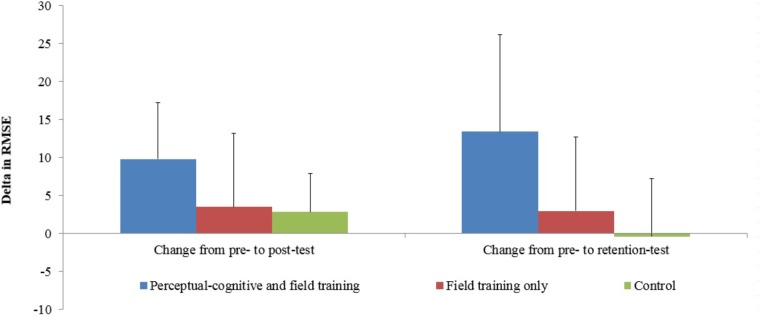
Comparison of changes from pre- to post-test (left bars) and from pre- to retention-test (right bars; bars indicate means while the lines above reflect standard deviations).

Our first hypothesis proposed a significant interaction between groups and pre- and post-test performance. An analysis of variance with groups as the between subject factor and pre- and post-test as the repeated measure revealed no differences between groups, *F*_(2,23)_ = 0.55, *p* = 0.59, but significant changes between tests, *F*_(1,23)_ = 11.03, *p* < 0.01, *f* = 0.68. Interestingly, the interaction of both factors was not significant, *F*_(2,23)_ = 2.07, *p* = 0.15, *f* = 0.42, but the effect size was large. As can be seen in **Figure 2** and **Table 1**, the cognitive and field training group improved the most followed by the field training group and the control group.

**Table 1:** Comparison of performances in pre-, post-, and retention-tests differentiated by groups (means and SDs in pixels).

	Pre-test	Post-test	Retention-test
Cognitive and field training group	67.54 (10.11)	57.73 (9.49)	54.14 (7.83)
Field training group	62.57 (9.97)	59.07 (9.57)	59.52 (9.69)
Control group	59.17 (8.02)	56.37 (12.08)	59.55 (12.21)

For our second hypothesis, we investigated changes from pre- to retention-test with the same analysis of variance approach. Again, no differences between groups were revealed, *F*_(2,23)_ = 0.91, *p* = 0.91; however, the repeated measure factor test, *F*_(1,23)_ = 6.20, *p* = 0.02, *f* = 0.51, and the interaction of both factors, *F*_(2,23)_ = 3.86, *p* = 0.04, *f* = 0.57, were significant. As can be seen in **Figure 2** and **Table 1**, the highest improvement was for the combined group.

## Discussion

In our first hypothesis we assumed a greater improvement in the cognitive and field training group compared to both other groups. While we did not find the expected significant interaction, the descriptive results were in the anticipated direction and the effect size was large. Based on these results, the augmented cognitive training seemed to be beneficial for improving pattern recall skills containing tactical elements. Additionally, the results related to our second hypothesis revealed that it also enabled better retention. Moreover, the long term effect (from pre- to retention-test) – as shown by the significant interaction – was larger than the short-term effect (from pre- to post-test; *f* = 0.42 vs. 0.57). These results indicate that augmented perceptual-cognitive skill training is beneficial for learning in the long-term.

These findings support previous research emphasizing the potential of perceptual cognitive training interventions (Put et al., 2013; Murgia et al., 2014). However, much of the prior work in this area has been done with novel training paradigms that are disconnected from athletes’ actual training environments (i.e., how the intervention interacts with an athlete’s regular training is unknown). In the current study, we tested an intervention that ran in parallel with athletes’ actual on-field training. This allowed us to determine the applicability of a perceptual cognitive intervention as an augmentation to regular training.

While these results provide some promising initial results, several limitations must be noted. First, future work is necessary to verify whether these laboratory results transfer to field performance. This is a consistent limitation of much of the research in this area (for exceptions see Christina et al., 1990; Harle and Vickers, 2001) and while we acknowledge the difficulty of field testing a pattern recall task, future studies should try to implement field tests. A second concern relates to the potential value of a placebo group. Although a placebo group offers a nice method of experimental control, in the current study we had to ensure that all athletes received the same training and dividing the team into yet another group was not feasible. A third concern relates to understanding the precise value of pattern recall to expert perception and anticipation. Looking at the role and mechanisms underpinning recognition of patterns and anticipation of experts, North et al. (2011) demonstrated that anticipation as well as recognition tasks stimulate complex memory structures and representations. Nevertheless, the activated memory representations during recognition tasks differ from the level of cognitive processing during anticipation tasks (North et al., 2009, 2011). Furthermore, Gorman et al. (2015) showed significant differences in visual search strategies in pattern recall and decision-making tasks, suggesting that solving these tasks requires the use of different underlying mechanisms. However, the role and mechanisms underpinning recall of patterns have not been clarified. On the surface, the role of being able to identify complex patterns of domain specific information for individual performance is not immediately clear. It is possible that it plays some role in search and retrieval of domain specific information that facilitates rapid decision-making and/or anticipation, however, further work is necessary to determine the precise role pattern recall plays in expert perceptual-cognitive performance.

This study highlights several areas for further work. First, our sample consisted of good, but not excellent youth players. Future work should examine whether these results apply to players with a higher level of skill and/or age. Second, an intriguing future area would be to test differences in the retention period. While our study had an unfilled retention phase, comparing either field-based or laboratory-based retention periods might provide helpful information for optimizing training plans. This study represents an important step in bridging the gap between laboratory-based perceptual learning studies and applied on-field training of athletes. Clearly more steps are necessary; however, continued research in this area would clarify the value of augmented video training for skill acquisition and expertise development.

## Ethics Statement

We do not have an approval from an ethical committee, because the study was conducted as a master thesis and no approval was needed at the time of conduction.

## Author Contributions

JS and JH designed the study. LF programmed the test. JH collected the data. JS and JB drafted the first version of this manuscript. JS, LF, MS, and JB revised and discussed all versions of this manuscript. All authors agreed on this latest version of the manuscript.

## Conflict of Interest Statement

The authors declare that the research was conducted in the absence of any commercial or financial relationships that could be construed as a potential conflict of interest.
